# Mitochondrial Dysfunctions and Potential Molecular Markers in Sport Horses

**DOI:** 10.3390/ijms23158655

**Published:** 2022-08-04

**Authors:** Agnieszka Dzięgielewska, Aleksandra Dunislawska

**Affiliations:** Department of Animal Biotechnology and Genetics, Bydgoszcz University of Science and Technology, Mazowiecka 28, 85-084 Bydgoszcz, Poland

**Keywords:** care, gene expression, horse management, mitochondria, mitochondrial genome

## Abstract

Mitochondria are an essential part of most eukaryotic cells. The crucial role of these organelles is the production of metabolic energy, which is converted into ATP in oxidative phosphorylation. They are also involved in and constitute apoptosis, the site of many metabolic processes. Some of the factors that negatively affect mitochondria are stress, excessive exercise, disease, and the aging process. Exercise can cause the release of large amounts of free radicals, inflammation, injury, and stress. All of these factors can contribute to mitochondrial dysfunction, which can consistently lead to inflammatory responses, tissue damage, organ dysfunction, and a host of diseases. The functions of the mitochondria and the consequences of their disturbance can be of great importance in the breeding and use of horses. The paper reviews mitochondrial disorders in horses and, based on the literature, indicates genetic markers strongly related to this issue.

## 1. Introduction

Years of evolution and improvement of the performance characteristics of horses have led to excellence in sports use. However, vigorous physical activity leads to various changes and adaptations that can affect the health and performance of horses. Key changes in the organism occur through loss of fluid and electrolytes, mainly through sweating and ventilation, which leads to the accumulation of harmful waste products and, consequently, to cell damage. These changes lead to the appearance of symptoms of fatigue, exhaustion, and stress [1]. Therefore, the assessment of changes that occur in the horse’s organism after physical exertion can help to estimate its competence to the expected workload, help to obtain information on changes caused by stress and fatigue, and also enable better prevention and treatment of injuries resulting from overexertion, which can significantly improve the welfare of these animals. Mitochondria play an important role during physical exertion. These organelles are enzyme-rich structures that catalyze the oxidation of organic nutrients by oxygen molecules, producing carbon dioxide and water. During these oxidation processes, chemical energy is released and is used to produce adenosine triphosphate (ATP), the most important energy carrier molecule in the cell. ATP synthesized by mitochondria spreads throughout the cell and is used for many cellular functions [2]. Mitochondria are the main site for generating reactive oxygen species (ROS), an excess of which can be harmful to the cell. The damage induced by ROS and reactive nitrogen species within the mitochondrial genome is the source of mitochondrial mutagenesis. The increase in the destruction of molecular cell structures contributes to many biological consequences, including changes in gene expression, mutations, weakened intercellular communication, tissue disorientation, organ dysfunction, or increased susceptibility of the organism to stress [3]. Destruction, inhibition, or disruption of mitochondrial metabolism may lead to the occurrence of all possible complications in the organism [4]. Literature data show that physical exercise increases inflammation mediators, reactive oxygen species, and markers of muscle damage initiated by intense training. This review aimed to identify possible sources of mitochondrial disorders in sport horses and to select potential molecular markers related to this process.

## 2. Mitochondria and Mitochondrial Genome

Mitochondria are semi-autonomous cytoplasmic organelles, spherical or elongated in shape, surrounded by two membranes [2], and found in most eukaryotic cells. They are characterized by a high degree of compartmentalization [5] and contain the mitochondrial genome in the form of a small circular DNA molecule, a set of specific mitochondrial RNAs, and ribosomes involved in synthesizing some mitochondrial proteins [6]. They are similar in size to bacteria and their number can be as high as approximately 2000 per cell [7]. Mitochondria are the site of cellular respiration and are responsible for over 90% of energy production in mammals [8]. The greatest number of mitochondria are found in cells in the myocardium and skeletal muscles, which require a lot of energy. Most organs, e.g., the liver are responsible for detoxification and the brain [9]. Mitochondria are surrounded by a double-membrane system composed of outer and inner mitochondrial membranes [10]. Both mitochondrial membranes form two mitochondrial compartments: the matrix as a large internal space and the intermembrane space which is a very narrow space [7]. The outer membrane is smooth and highly permeable to small, water-soluble molecules such as ions, sugars, and amino acids [5]. It contains wide water channels formed by porins—transport proteins. It also includes enzymes needed for synthesizing mitochondrial lipids and enzymes that transform lipid substrates. The inner mitochondrial membrane creates numerous folds that penetrate deep into the mitochondrion, increasing the area of cellular respiration. Unlike the outer membrane, it is not permeable to ions and most small molecules, making its contents highly specialized—it contains only those molecules that will be selectively transported across the inner membrane [7]. This is essential for maintaining the proton gradient that drives oxidative phosphorylation [10]. In the inner membrane, there are proteins responsible for carrying out the oxidation reaction through the electron transport chain, the production of ATP by ATP synthase, and the transfer of metabolites to and from the matrix [7]. Currently, the theory of endosymbiosis is the most popular hypothesis explaining the origin of mitochondria. It is believed that mitochondria evolved from bacteria engulfed by primitive eukaryotic cells with which they initially lived in an endosymbiotic relationship [7]. The protomitochondrion created by endosymbiosis has rapidly evolved to adapt to the prevailing conditions inside the host cell. One of the stages was the transfer of genes from the protomitochondrial genome to the cell nucleus. This process led to a gradual reduction in the mitochondrial genome and an increase in the dependence of mitochondria on the nuclear genome. The production of metabolic energy in eukaryotic cells is a key role for the mitochondria. They are responsible for producing most of the useful energy from the breakdown of carbohydrates and fatty acids, which are converted into ATP in oxidative phosphorylation [11,12]. For this function to be fulfilled, many processes must occur within the mitochondria. These include pyruvate decomposition, the citric acid cycle, the respiratory chain, oxidative phosphorylation, β-fatty acid oxidation, the onset of gluconeogenesis, and the synthesis of ketone bodies. Other cellular processes in which mitochondria play an important role include apoptosis and cell type-specific functions. These organelles are involved in the metabolism of cholesterol, amino acids, and organic acids, oxidize fatty acids, synthesize sex steroids and heme, and also detoxify ammonia and metabolize neurotransmitters [13]. According to Kirtszenbaum and Tres [5], mitochondria also influence thermogenesis. Much of the oxidation energy is dissipated as heat and not converted to ATP. This is due to the participation of uncoupling proteins (UCP), belonging to the superfamily of mitochondrial anion carrier proteins. They are found in the inner mitochondrial membrane and mediate the regulated release of H^+^ with a consequent release of heat. As mentioned earlier, mitochondria regulate the process of programmed cell death. They contain apoptogenic factors in the intermembrane space, including cytochrome c, Apoptosis-Inducing Factor (AIF), procaspases, Smac/DIABLO protein (Second Mitochondria-Derived Activator of Caspases/Direct IAP Binding Protein of Low Pi), Omi/HtrA2 protein (High-temperature requirement protein A2) and endonuclease G [14]. In the presence of apoptotic signals, they are released into the cytoplasm, and some of them participate in the activation of caspases [2]. Cytochrome c, a component of the mitochondrial electron transport chain, is involved in the production of ATP and triggers caspase cascades. The apoptotic pathway can be activated when cytochrome c is released from the mitochondria into the cytoplasm. It is assumed that mitochondrial DNA (mtDNA) is also released into the cytoplasm. The leakage of cytochrome c and mtDNA takes place with the help of pro-apoptotic proteins Bax and Bak, which create an opening in the outer mitochondrial membrane that carries the mitochondrial components to the cytoplasm. Cytoplasmic cytochrome c, soluble mesothelial proteins, and procaspase 9 bind to Apoptotic Protease Activating Factor 1 (APAF1) to form a complex called the apoptosome. It conditions the activation of caspase 9, which is the initiator of apoptosis, and leads to the activation of caspase 3 and caspase 7. Consequently, all these proteases cleave several substrates inside the cell, which accelerates cell death [5].

MtDNA is genetic material found in the mitochondrial matrix, usually in the form of a circular, double-stranded molecule that is not associated with histones. However, several organisms in which mtDNA occurs in a linear form have been identified. The mitochondrial genome is polyploid, i.e., it consists of multiple copies. There is a discrepancy in the number of mtDNA copies between different types of cells and tissues and over the lifetime of the cell, resulting from the changing number of mitochondria throughout the life of each cell, the presence of multiple copies of the mitochondrial genome in the cell and the uncoordinated number of mtDNA copies directly with the cell cycle [13]. About 1300 complete mitochondrial genome sequences are known, many of which are animal genomes, but some genomes of higher plants are also known. The main difference between the mitochondrial genomes of animals and plants is their size and, to a lesser degree, their variability. In animals, mitochondrial genomes do not differ significantly in the set of genes and size, while in plants, they are characterized by both large size and variability, even within one species. A typical animal’s mitochondrial DNA has about 37 genes. In higher plants, this number usually ranges between 50 and 60 genes [15]. The mtDNA contains information about 22 types of tRNA, two types of ribosomal RNA molecules, and 13 proteins involved in the process of oxidative phosphorylation [16], among which Chowdhary et al. [8] list NADH1, NADH2, NADH3, NADH4L, NADH4, NADH6, NADH5, NADH5, COXI, COXII, COXIII, CYTB, ATP6, and ATP8. All these proteins are part of five protein complexes. The remaining proteins in the respiratory chain, enzymes related to DNA replication, translation, and transcription, and ribosomal proteins are encoded in the nuclear genome. They penetrate the mitochondria only after they have been transcribed in the nucleus and translated into the cytoplasm [17]. In the horse, the mitochondrial genome is 16,660 bp long. It contains a highly variable control region of about 1192 bp, containing up to 29 repeats of the GTGCACCT sequence, often showing heteroplasmy [8], a condition in which the body has more than one mitochondrial genome. The appearance of heteroplasmia in animals has been associated with mitochondrial diseases and the aging process, but it has also been detected in healthy people [18]. A high density of coded sections characterizes MtDNA. The only fragment devoid of genes is the so-called loop D, which is the site of transcription initiation. There are two smaller units called hypervariable areas. Both regions are non-coding sequences [19]. A separate DNA polymerase, called gamma DNA polymerase, which is located in the mitochondria [12], is responsible for mtDNA replication. It is encoded by the nuclear protein POLG (DNA Polymerase Gamma, Catalytic Subunit), the mutations of which may disrupt the function of a specific endonuclease, consequently leading to somatic mutations in the mtDNA that persist in future replication. Another important feature of mtDNA replication is the reduced stringency of the correction of replication errors, consequently leading to greater sequence variability compared to the nuclear genome, which may have a phenotypic or pathogenic effect. However, mutational events are the only source of genetic variation in the mitochondrial genome because it does not recombine, unlike homologous pair recombination [20]. The variability of the mitochondrial genome is regulated by several important features. These include non-Mendelian maternal inheritance, non-recombination, a faster rate of evolution, and a lower effective population size. There are more base pair variants in the mitochondrial genome compared to the nuclear loci. This is due to the faster evolution of the mtDNA sequence compared to the nuclear genome. For this reason, the mtDNA genome is not always useful for reconstructing the history of the population because mitochondrial genes are inherited as one unit, and despite sequencing studies of the entire mitochondrial genome, only the origin of one locus is visible [8]. The mitochondrial genome is mainly responsible for coding over a dozen complex units located in the inner mitochondrial membrane. The rest of the subunits of these complexes are encoded in the cell nucleus and synthesized in the cytoplasm. Therefore, an important element of gene expression in mitochondria is to ensure an appropriate ratio between the number of mtDNA-encoded subunits with the number of cytoplasm-derived subunits. The main control in animal mitochondria is through the initiation of transcription. It is regulated by various factors, including TFAM, TFB1M, TFB2M, and mTERF. Another important process for the biogenesis of high molecular weight mitochondrial complexes is protein degradation. Protease ATP-dependent enzymes are involved in the removal of non-functional proteins or excess proteins. It is a specific process, i.e., a given type of protease has specific substrates [21].

There is ample evidence supporting the hypothesis that the mitochondrial genome arose within the eubacterial rather than archaeological domain of life. α-proteobacteria are the most closely related to mitochondria, as evidenced by, for example, phylogenetic analyzes of genes encoding proteins and ribosomal RNA (rRNA) genes determined by mtDNA. This thesis confirms that ATP production in combination with electron transport and translation of mitochondrial proteins constitutes the essence of mitochondrial function. These functions are common to all mitochondrial genes and can be unequivocally deduced from the α-proteobacterial ancestor. The mitochondrial genome encodes the necessary components for both processes [22]. In the evolutionary line of animals, there is a tendency to reduce the size of the mitochondrial genome and its maximum packing, which is associated with a small number of genes and the elimination of unnecessary mtDNA sequences [23].

## 3. Mitochondrial Disorders

Impaired functioning of the mitochondria and its inefficiency has a direct impact on the reduction in muscle efficiency, loss of concentration, memory, vision, and immunity impairment, as well as more frequent occurrence of hearing problems and all inflammatory processes in the body. It has been shown that many factors can negatively affect mitochondria. These include stress, excessive physical and mental exercise, diseases, taking medications, and the aging process [24]. During training, large amounts of free radicals are released by many tissues. The action of these molecules is the main cause of damage to the mitochondria. Free radicals and antioxidants neutralizing them are chemical compounds naturally occurring in organisms, necessary for proper functioning. Sometimes there is an imbalance between oxidants and antioxidants. This leads to a phenomenon called oxidative stress’ the body’s ability to remove ROS and repair the damage caused by overproduction is disrupted. The consequence may be damage to biological material—ordered molecular structures consisting of many atoms, such as nucleic acids, fats, and proteins. The cause of the excess of ROS is their excessive production in hyperoxia or hypoxia, or when their removal is limited, resulting from the reduction in enzymes catalyzing the metabolism of ROS, a shortage of antioxidants or glutathione [4]. Free radicals are highly active substances that form biologically unpredictable chemical bonds. They trigger chemical reactions whose products have no biological use. This results in the appearance of substances unnecessary to the body or destroyed cells. Over time, they can accumulate and lead to disturbances in the body’s functioning [25]. Free radicals can also threaten biological, cellular, or mitochondrial membranes, which are responsible for the active transport of substances and the creation of a specific environment, the balance of which is an indispensable condition for chemical processes. Oxidative stress causes the mitochondrial permeability transition (MPT) to open up and the ATP count to drop. If the oxidative damage is minor, it is eliminated with the help of autophagy and mitophagy, which enables the cells to survive. As a result of oxidative damage to mitochondria, there is an increased tendency of mitochondria to release proteins in the intermembrane space, e.g., cytochrome c to the cytosol through the permeability of the outer membrane of the mitochondria, which in turn triggers the process of programmed cell death [26]. In a critical situation, the outer mitochondrial membrane ruptures when oxidative stress reaches an extreme level. This results in irreversible swelling and necrosis. Both these factors, together with the infection, lead to the release of the High Mobility Group Box 1 protein (HMGB1) [9]. In addition, the production of free radicals leads to the induction of permeability transition pores (PTP), which results in the permeability of the inner membrane for small molecules in situations such as ischemia-reperfusion. Therefore, oxidative damage to the mitochondria is the cause of many pathologies. Moreover, mitochondrial ROS may act as a modulated redox signal that reversibly affects the activity of several functions in the mitochondria, cytosol, and nucleus [26]. Characteristic features of mitochondrial diseases are incomplete penetration, variable expression, and pleiotropy. The phenotypic expression of mitochondrial disorders depends on the proportion of normal and mutated mitochondrial genomes found in cells of various tissues. Therefore, the impairment of the function of a given organ and its degree of advancement depends on the percentage of mutated mitochondria. As mitochondria are present in the cells of the whole organism, they are responsible for the pathology of various organs and systems [27]. In mammals, mitochondrial DNA evolves and mutates several times faster than nuclear DNA. The causes of the mutation include less efficient mtDNA repair processes, and the formation of free radicals in the oxidation process. These causes can lead to DNA damage [28].

Because the mitochondrial genome does not recombine during fertilization, sperm do not contribute significant cytoplasmic components to the zygote, including mitochondria, and inherited mitochondrial disorders are transmitted in a matrilineal manner. This means that all children of the sick mother will inherit the disease, but it will not be passed on from the sick father. Changes in the mitochondrial genome that engage enzymes required for oxidative phosphorylation can lead to reduced ATP supply, generation of free radicals, and the induction of apoptosis. Several syndromic disorders caused by mutations in the mitochondrial genome are known in humans; they influence both protein-coding and tRNA genes. Due to the great dependence of brain and muscle structures on oxidative phosphorylation, a wide clinical spectrum often includes myopathies and encephalopathies. The clinical course and age of onset of the disease vary due to atypical mtDNA transmission mechanisms—replication independent of nuclear DNA [13]. A mutation in one of the genes of the mitochondrial tRNA is associated with metabolic syndrome, which manifests itself in obesity and diabetes. In turn, mutations in the mitochondrial genes encoding components of the electron transport chain can lead to Leber’s hereditary optic neuropathy, leading to blindness. In addition, it is believed that the gradual accumulation of mutations in mtDNA during the lifetime of a given organism contributes to the aging process [12], and acquired somatic mutations in the mitochondria are involved in several age-related degenerative diseases affecting mainly the muscles and nervous system. Sometimes, due to the high degree of polymorphism in mtDNA and phenotypic variability, it is difficult to determine whether a change in mtDNA is the cause of the clinical phenotype. Some pharmacological therapies may have a negative effect on mitochondria and their functions. For example, the appearance of acquired mitochondrial myopathy following azidothymidine treatment, which reduces the number of mtDNA in muscles, is given [13].

Mitochondria are very sensitive to stress. Long-term stress contributes to an increase in the frequency of inflammation, which in turn leads to an increase in the number of free radicals and nitric oxide (II), inhibition of sugar metabolism, disturbance of the balance of the endocrine system and the nervous system [29]. The prolonged load of nitric oxide (II), i.e., nitrosative stress, leads to many mitochondrial dysfunctions, including fibromyalgia, metabolic syndrome, neurodegenerative diseases, and rheumatic diseases of the locomotor system [30]. Stress hormones can interfere with the function of lymphocytes. T lymphocytes play an important role in regulating the body’s efficiency, and the presence of mitochondrial dysfunctions within them causes multi-disease and premature aging [9].

The main mechanism of mitochondrial quality control is the elimination of dysfunctional and damaged mitochondria by selective autophagy—mitophagy. Damaged mitochondria can be a source of mitochondrial components such as mtDNA with hypomethylated CpG patterns or cardiolipin. These molecules are then detected as danger-related molecular structures (called alarmins, or DAMP, or Danger/Damage Associated Molecular Patterns) and can trigger innate immune signaling [31]. Due to this release of DAMP molecules, mitochondria can direct the immune response towards inflammation. Conversely, if mitochondrial DAMPs are released by damaged cells without the presence of infection, they can induce unwanted inflammatory responses, which in turn may contribute to tissue damage and organ dysfunction [9].

## 4. Diseases Resulting from the Sport Use of Horses

Sports use of horses places high demands on strength and endurance, which may increase the risk of injury. The most common consequences are pain, lameness and poor performance, which make it difficult to move around properly. The loads that horses may struggle with may contribute to the occurrence of various orthopedic diseases [32]. The most common cause of such diseases is excessive physical effort during training or competition, consistently leading, among others, to traumatic fractures or fatigue fractures [33]. One of the reasons for injuries in horses may be the poor development of anatomical structures of the locomotor system, especially tendons in racehorse breeds. This is due to the early onset of excessive training in young horses, hereditary defects, and insufficient time to move. The rider’s skills, the type of ground, the condition of the horse [34], and its mental state as well as random situations in which accidents and sudden injuries occur [32]. Overloading, especially in young horses, can lead to sesamoiditis, osteochondroses, bone fractures, and fractures [35]. Fractures may occur in the hoof bone, calcaneus bone, wrist bone, metacarpal and metatarsal bones, hoof bone extension, sesamoid, patella, tibia, ulna, femur, and radius [32]. Excessively intensive work and failure to adjust the intensity of work to the horse’s age, condition, and advancement may result in arthrosis, i.e., chronic ankylosing arthritis, such as spar or frog. Failure to start training with warm-up and training overload may cause inflammation and tear of tendons or ligaments. Other diseases include chronic inflammation of the stifle joint caused by ossification of the meniscus, acute shoulder and stifle joint inflammation, calcification, laminitis and superficial purulent inflammation [32]. The psychological state of the animal may be a factor influencing the appearance of limb diseases in horses, e.g., stress disturbing homeostasis. Exercise is stressful for all animals, but the intensity of the stress response depends on the intensity and duration of exercise as well as the condition of the animal [36]. Sustainable exercise can determine the forms of adaptation that improve performance and can be called eustress. The stress hormone cortisol plays an important role in adaptation during exercise. It supports the regeneration of the locomotor system through increased protein synthesis and increases the rate of fat depletion through greater deposition and storage of glycogen. Therefore, an increased concentration of cortisol prepares the body for long-term stimulation. However, long-term exposure to stressors causes an inability to induce an adaptive response, which in turn leads to decompensation and puts the body into an exhaustion phase with various adverse outcomes. For this reason, chronic stress is considered to be the main threat to animal welfare. It has been found that many components of the human immune system are weakened by prolonged exercise [37,38,39]. Intense exercise may induce neutrophil degranulation, resulting in increased plasma concentrations of neutrophil marker enzymes—myeloperoxidase and elastase—in humans [40,41] and rats [42]. These enzymes have pro-inflammatory and pro-oxidative properties and may play a role in exercise-induced muscle damage. It was also noted that the increase in myeloperoxidase and elastase was correlated with the increase in creatine kinase, which is considered a marker of muscle damage [43]. The intensity of degranulation increases with increasing exercise intensity [44]. Additionally, leukocytes are involved in aseptic myositis associated with damage to muscle fibers after demanding exercise [43]. It has been shown that after vigorous exercise, both in humans and animals, the transfer of neutrophils into skeletal muscles increases the concentration of myeloperoxidase in the tissues [45,46]. It is believed that repeated injury to muscle fibers caused by strenuous exercise may result in a sustained systemic response to cytokines [47]. It may be associated with chronic inflammation, immune system dysfunction, overtraining syndrome [48], or unexplained underperformance syndrome [49]. Another study found high lactemia after exercise, which may be associated with mitochondrial dysfunction in musculoskeletal muscles [50].

## 5. Markers Potentially Associated with Mitochondrial Dysfunction in Horses

Myostatin (MSTN) is one of the main genetic determinants of the type of muscle fibers, it is related to strength and endurance [51]: it inhibits the proliferation and differentiation of myoblasts in muscle development [52], which limits muscle growth and is produced mainly in skeletal muscles [53]. Therefore, inactivation or inhibition of myostatin promotes muscle growth [54]. However, studies in mice have found that mice deficient in MSTN have fewer mitochondria, consequently leading to changes in skeletal muscle activity and enzyme capacity. A reduced number of mitochondria and their activity may affect skeletal muscle function by impairing mitochondrial metabolism and reducing the contractile capacity of the muscle [55].

As shown in the literature, the activity of citrate synthase (CS) is strongly correlated with the content of mitochondria [56,57], which is usually the largest in type I muscles (slow-twitch, oxidative), smaller in type IIa muscles (fast-twitch, oxidative), and the smallest in type IIx muscles (fast-twitch, glycolytic) [57]. It has been observed that training from around the age of two causes an increase in the activity of oxidative enzymes in skeletal muscles and increases in fibers with high oxidative activity. Along with the increase in the activity of oxygen enzymes, the volume density of the mitochondria increases. Increasing the oxidative capacity influences the body’s efficiency due to the increased efficiency of ATP generation, which delays the accumulation of lactate in the muscles and blood during anaerobic exercise. It reduces the use of glycogen (in favor of free fatty acids) during aerobic exercise [58].

Strenuous exercise can affect neutrophil degranulation, which in turn contributes to an increase in the plasma concentration of neutrophilic markers such as myeloperoxidase (MPO) and elastase (ELANE). This degranulation tends to increase with increasing exercise intensity [59]. Activated neutrophils generate ROS and release oxidizing enzymes as well as ELANE and MPO proteases. Moreover, MPO has been shown to significantly increase the amount of ROS generated by anoxic and reoxygenation cycles. Therefore, the production of ROS can be explained by the activation of neutrophils with the presence of MPO and/or by mitochondrial dysfunction [47]. It was found that the increase in MPO and ELANE content was correlated with the increase in plasma and muscle creatine kinase (CK) levels, which may suggest the involvement of these enzymes in muscle damage [60]. Stress-induced muscle damage leads to the production of myoglobin and CK, which mobilize neutrophils into the housing of the muscle tissue. This is a protection mechanism against inflammation, infection, and oxidative stress [61]. For this reason, CK is a good marker of the degree of training advancement, as its increased expression may indicate the appearance of microdamage in the muscle fibers [53].

Erythropoietin (EPO) is a peptide hormone produced by the kidneys in response to hypoxia sensed by cells located in the vessels of the renal matrix. When cardio-respiratory regulation is inadequate, and factors such as blood loss or strenuous exercise are large enough to cause the partial pressure of oxygen in the renal arteries to drop, plasma EPO levels increase, and erythropoiesis is stimulated [58]. Local hypoxia is an important stimulus for structural and functional changes in skeletal muscle. The major regulator of the hypoxia response, HIF-1a, regulates the oxidative enzyme COX4 in an oxygen-dependent manner by alternating the recruitment of the COX4I1 and COX4I2 isoforms [62]. At normal oxygen concentrations, the expression of the COX4I2 gene is repressed, while lowering the oxygen concentration results in the degradation of the COX4I1 protein with a simultaneous increase in the activity of HIF-1, LONP1, and COX4I2. This switching of COX2 subunits provides a mechanism for maximizing respiratory efficiency at various oxygen concentrations [63]. Therefore, an increase in COX4I2 is observed after vigorous exercise and hypoxia [64]. Whereas another cytochrome c oxidase—COX-2 is activated in response to a variety of extracellular or intracellular physiological stimuli. Overexpression of COX-2 metabolizes the accumulation of prostaglandins E2 (PGE2). PGE2 target molecules mobilize several signaling pathways and reduce the level of apoptotic proteins, thereby contributing to a variety of physiological processes, including proliferation, angiogenesis, and metastasis. Growth and overexpression of COX-2 are mainly associated with inflammation, loss of apoptosis, uncontrolled cell proliferation, growth, metastasis, neovascularization, and angiogenesis, ultimately leading to cancer. Prostaglandins generated by COX-2 also act as immunosuppressants. It has been shown that macrophage and NK cell-mediated cytotoxicity is suppressed by PGE2 [65].

As shown in the literature, the lack of ACTN3 in horses can cause a rapid reduction in fiber diameter, number, and the surface of type II muscle fibers, an increase in the activity of many enzymes, and faster regeneration after exercise. On the other hand, the presence of the SNP ACTN3 R577X polymorphism in this gene, which results from the replacement of arginine in codon 577 with the STOP codon, is associated with exercise-induced muscle damage [53].

Because the enzyme LDH is present in many cells, the total concentration of LDH increases when cells are damaged. This concentration increases in all disease states during which tissue necrosis occurs, including acute damage to the heart muscle, red blood cells, or skeletal muscles [66]. The transcription factors regulating the response to intense physical exercise include, among other things, NRF1 and SRF which regulate the expression of TFAM, respiratory and mitochondrial protein transporters, and muscle growth [67]. Intensive training significantly influences mitochondrial biogenesis, which PPARGC1A and PPARD [53] largely regulate. The PPARD protein is a transcription factor that plays the role of a central regulator of the expression of genes encoding proteins involved in lipid metabolism, especially in the metabolic pathways of beta-oxidation in the mitochondria. PPARGC1A directly connects external physiological stimuli with mitochondrial biogenesis, regulates the type of muscle fibers, and is associated with endurance exercise.

Additionally, it mediates the regulation of insulin secretion and regulates oxidative energy metabolism during exercise in the skeletal muscles of horses. For this reason, it is involved in the adaptation of horses’ skeletal muscles to training [51]. As shown in the literature, AMPK is responsible for reducing energy availability by reducing the activity of energy-requiring reactions, such as protein and lipid synthesis and the cell cycle. In addition, it improves energy production thanks to increased catabolism and modulates basic mitochondrial processes, including biogenesis or autophagy, and promotes mitochondrial health [68]. AMPK regulates energy metabolism by sensing energy deficiency. Activation of AMPK promotes ATP-producing metabolic pathways and inhibits ATP-consuming pathways [9]. During metabolic stress, ATP synthesis slows down, which then leads to a faster change in the AMP/ATP ratio compared to ADP/ATP, resulting in the activation of AMPK by the presence of AMP. Moreover, it was observed in human studies that the formation of ROS and inhibition of the activity of the mitochondrial respiratory chain influence the activation of AMPK [69].

In many studies, muscle response to exercise has been assessed using the activity of mitochondrial enzymes such as SDH, by studying the muscle capillary supply and determining the volume density of the mitochondria. Additionally, SDH is considered to be the main source of H_2_O_2_ [70]. Uncoupling proteins, including UCP2, are an important control point for the cell’s energy management. They are responsible for dissipating the useful free energy derived from the oxidation of respiratory substrates. Their important function is to protect the cell against oxidative stress by reducing the production of ROS [71].

The increase in TFAM protein provides for the coordinated induction of mtDNA transcription and replication, leading to increased expression of mitochondrial proteins essential for respiratory chain function. In cell culture studies, the contractile activity-induced increases the expression of TFAM, NRF-1, and PGC-1 proteins. These changes provide a favorable environment for the initiation of mitochondrial biogenesis in muscle cells. This is because PGC-1 coactivates NRF-1, which transcriptionally activates the promoters of nuclear genes encoding mitochondrial proteins, including the important mtDNA transcription factor, TFAM [72]. However, TFAM has been found to play an important role in the process of induction of polymorbidity and premature aging by T cells with dysfunctional mitochondria [9]. In the early stages of muscle regeneration and repair, FN1 levels increase, allowing mitotic satellite cell adhesion, muscle fusion, and regeneration. Inactive satellite cells can respond to mechanical stimuli and damage by becoming activated, differentiating into myoblasts, which can bind together to regenerate lost tissue or fuse with existing fibers to allow the repair of muscle fibers [73].

Based on the literature, a list of expected directions of changes in gene expression was prepared (Table 1), which can be considered markers for conditions caused by mitochondrial dysfunction in horses.

## 6. Conclusions

Exercise in horses may contribute to the increase in MPO levels, which is associated with the state of increased oxidative stress and inflammation. Its dysregulated release can lead to tissue damage. MSTN induces mitochondrial metabolic changes. A decrease in its concentration was observed in the post-workout state. Loss of MSTN function can lead to decreased mitochondrial content, decreased expression of cytochrome c oxidase, and lower citrate activity in skeletal muscle.

Moreover, hypertrophic muscles deficient in MSTN show severe fatigue associated with abnormal mitochondrial and lipid metabolism. A link was found between CK activity and changes in oxidative phosphorylation, which may suggest a relationship between exercise-induced muscle damage and a decline in mitochondrial respiration. ACTN3 may alter CK activity and increase the skeletal muscle injury response to exercise. The increase in AMPK concentration may indicate the presence of oxidative stress and metabolic stress, i.e., the accumulation of secondary metabolites in muscles undertaking the increased effort. The increase in ELANE following muscle injury induced by vigorous exercise may reflect the increased activity of neutrophils associated with inflammation. In addition, MPO-mediated ROS generation may provide a favorable environment for ELANE activity due to the inactivation of its inhibitors. It is believed that chronic exercise may lead to the dysregulation of erythropoiesis through a chronic increase in EPO. Disturbed erythropoiesis may indicate impaired mitochondrial fusion, which results from the key role of mitochondria in the differentiation of hematopoietic stem cells. It is assumed that mitochondria produce ROS by leakage of single electrons in the respiratory chain in the mitochondrial inner membrane. There are many sites of H_2_O_2_ formation in the mitochondria, one of them being complex I of the electron transport chain, i.e., NADH dehydrogenase. SDH being one of the main sources of H_2_O_2_ indicates its possible participation in oxidative stress. Low expression of UCP2 may increase the accumulation of ROS in the mitochondria and may be responsible for apoptosis and necrosis in muscle fibers. Mitochondrial proliferation in pathological conditions is sometimes associated with an increase in CS activity. After exercise, there is a correlation between increased COX4I2 gene transcription and increased mitochondrial density, suggesting exercise-induced mitochondrial plasticity. In response to acute energy demands, PGC-1α (encoded by the PPARGC1A gene) locates in the nuclear and mitochondrial compartments to act as a co-activator of the transcription of DNA transcription factors. This may suggest that PGC-1α facilitates nuclear and mtDNA penetration to promote mitochondrial biogenesis. PPARD is one of the receptors that play a major role in metabolism and inflammation control. Exercise can modulate the expression of this gene, including in skeletal muscles. It is needed to maintain metabolic homeostasis and the anti-inflammatory effect of acute exercise. Its lack may induce the overexpression of pro-inflammatory cytokines. NRF1 is an important regulator of the nucleus-encoded mitochondrial subunits of the respiratory complexes, and its disruption can lead to a significant loss of mtDNA. Exercise induces complex I to fold into supercomplexes in skeletal muscle. This phenomenon is inversely related to oxidative damage to the mitochondria. Super complexes may contribute to the systemic antioxidant activity of exercise. Lack of TFAM can lead to the appearance of abnormal mitochondria in the muscles and the progressive deterioration of respiratory chain function, as well as a reduction in mtDNA copy number and mitochondrial encoded protein content. The loss of FN1 leads to ineffective muscle remodeling. When this happens, satellite cells are unable to attach to FN1, leading to cell death by anoikis (a type of apoptosis). Exercise inhibits COX-2 activity, leading to the suppression of pro-inflammatory cytokines and changes in the redox state.

## Figures and Tables

**Table 1:** Selected genetic markers related to the mitochondrial disorders in horses with the expected direction of changes in gene expression—markers associated with mitochondrial disorders observed by other authors.

Marker Name	Gene Symbol	Functions	Possible Direction of Changes	References
ADP/ATP Carrier	*SLC25A4, SLC25A5, SLC25A6, SLC25A31*	a specialized transport protein for the export of mitochondrial ATP into the cytoplasm for energy delivery to the cell; its deficiency or dysfunction contributes to serious consequences for cellular metabolism and may lead to various diseases, including muscular dystrophy; it plays a role in programmed cell death and cancer [74]	↑ *SLC25A4* ↑ *SLC25A5*	[51,75]
Myostatin	*MSTN*	a negative regulator of muscle growth and an inhibitor of satellite cell proliferation [75]	↓	[51,75]
Citrate synthase	*CS*	an enzyme involved in the Krebs cycle; it is located in the mitochondrial matrix; it is nuclear-encoded, synthesized on cytoplasmic ribosomes, and then transported to the mitochondrial matrix; commonly used as a marker of mitochondrial content [76]	↑	[57,75,77,78,79,80]
Myeloperoxidase	*MPO*	heme peroxidase is considered to be a marker of neutrophil activation in the inflammatory process [59,81,82]	↑	[59,60,83]
Elastase	*ELANE*	an inflammatory neutrophilic enzyme; it is released by activated neutrophils and is considered an inflammatory and prognostic marker in various diseases; involved in inflammatory tissue damage and plays a role in ischemia-reperfusion injuries [59]	↑	[43,59]
Erythropoietin	*EPO*	together with *PPARD* and *UPC*, it is a group of major genes associated with maximum oxygen uptake [53]	↑	[84]
Creatine kinase M-type	*CKM*	an enzyme that catalyzes the donation of phosphate from creatine phosphate to ADP; it is found in skeletal muscle, heart, and brain [38]; increased concentration of CK is a characteristic symptom of muscle damage caused by intense exercise [83]	↑ ↓	[63,75,82,83,85]
Cytochrome c oxidase subunit 4 isoform 2	*COX4I2*	nuclear-encoded regulatory subunit in the mitochondrial terminal complex of the electron transport chain; plays a significant role in the inflammatory response and oxidative stress [86]	↑	[63,87]
Alpha-actin-3	*ACTN3*	involved in muscle structure [88]; plays a key role in the rapid action of muscle fibers [53]	↓	[75]
Lactate dehydrogenase (LDH)	*LDHA*	an enzyme found in the cells of many tissues, including skeletal muscle, heart, brain, liver, red blood cells, and lungs; is responsible for the conversion of lactic acid to pyruvic acid in the presence of NADH in the muscles [66]	↑ ↓	[63,66,80,89]
Succinate dehydrogenase (SDH)	*SDHC*	encodes one of four nuclear-encoded subunits that include succinate dehydrogenase; its activity is used as a marker of oxidative muscle capacity [50,63]	↑	[50]
Mitochondrial transcription factor A	*TFAM*	controls the transcription of mitochondrial proteins [88]	↑	[72,90,91]
Mitochondrial uncoupling protein	*UCP2*	it enables a controlled “leakage” of protons from the intermembrane space to the mitochondrial matrix [71]	↓	[51,84]
5′AMP-activated kinase (AMPK)	*PRKAA1*, *PRKAG3*	energy metabolism marker; activity is modulated mainly by the AMP/ATP ratio and to a lesser extent by the ADP/ATP ratio, which are direct biomarkers of the state of energy availability [68]	No data	No data
PPARG coactivator 1 alpha	*PPARGC1A*	plays the role of the main regulator of mitochondrial biogenesis and activates mitochondrial transcription factors [53]	↑	[51,63]
peroxisome proliferator-activated receptor	*PPARD*	marker of maximum oxygen uptake [53]	↑	[51]
Fibronectin 1	*FN1*	involved in cell adhesion, migration, growth, and differentiation; the marker of damage to muscle fibers that may occur after intense physical exercise [51]	↑	[51,84]
NADH dehydrogenase	*ND2*	one of the mitochondrial enzyme complexes involved in the association of electron transfer with proton translocation from the mitochondria, which allows the generation of a transmembrane proton driving force that drives ATP synthesis [92]	↓	[84]
Prostaglandin-endoperoxide synthase 2	*COX2*	one of the cytochrome oxidase subunits; involved in oxygen reduction	↓	[84]
Nuclear respiratory factor 1	*NRF1*	a marker of mitochondrial biogenesis [93]; involved in the regulation of metabolism, cell growth as well as mtDNA transcription and replication [53]	↑	[90,94]

## Data Availability

Not applicable.
